# Thermodynamic Assessment of the P_2_O_5_-Na_2_O and P_2_O_5_-MgO Systems

**DOI:** 10.3390/ma17102221

**Published:** 2024-05-08

**Authors:** Lideng Ye, Chenbo Li, Jifeng Yang, Guangcheng Xiao, Zixuan Deng, Libin Liu, Ligang Zhang, Yun Jiang

**Affiliations:** 1 School of Material Science and Engineering, Central South University, Changsha 410083, China; 2 NMPA Key Laboratory for Pharmaceutical Excipients Engineering Technology Research, Hunan Institute for Drug Control, Changsha 410001, China

**Keywords:** P_2_O_5_-Na_2_O system, P_2_O_5_-MgO system, CALPHAD, thermodynamic optimization, phase diagram

## Abstract

Knowledge about the thermodynamic equilibria of the P_2_O_5_-Na_2_O and P_2_O_5_-MgO systems is very important for controlling the phosphorus content of steel materials in the process of steelmaking dephosphorization. The phase equilibrium and thermodynamic data of the P_2_O_5_-Na_2_O and P_2_O_5_-MgO systems were critically evaluated and re-assessed by the CALPHAD (CAlculation of PHAse Diagram) approach. The liquid phase was described by the ionic two-sublattice model for the first time with the formulas (Na^+1^)_P_(O^−2^, PO_3_^−1^, PO_4_^−3^, PO_5/2_)_Q_ and (Mg^+2^)_P_(O^−2^, PO_3_^−1^, PO_4_^−3^, PO_5/2_)_Q_, respectively, and the selection of the species constituting the liquid phase was based on the structure of the phosphate melts. A new and improved self-consistent set of thermodynamic parameters for the P_2_O_5_-Na_2_O and P_2_O_5_-MgO systems was finally obtained, and the calculated phase diagram and thermodynamic properties exhibited excellent agreement with the experimental data. The difference in the phase composition of invariant reactions from the experimentally determined values reported in the literature is less than 0.9 mol.%. The present thermodynamic modeling contributes to constructing a multicomponent oxide thermodynamic database in the process of steelmaking dephosphorization.

## 1. Introduction

As society progresses, industries advance to higher developmental stages, leading to more demanding usage of steel materials and increased quality requirements for steel materials across various sectors. Phosphorus, as one of the detrimental elements in steel, serves as a critical indicator of steel quality. Therefore, the control of phosphorus content in steel remains a crucial target for enterprise development. In the steelmaking process, the inclusion of alkaline earth metal oxide fluxes such as MgO can effectively diminish phosphorus in liquid steel, while alkali metal oxide fluxes like Na_2_O also exhibit a strong dephosphorization effect [1]. The phase diagrams and thermodynamic properties of the P_2_O_5_-Na_2_O and P_2_O_5_-MgO systems are essential to effectively control the dephosphorization effect of slags and to understand the phosphorus distribution ratio between liquid iron and oxide slags such as Na_2_O and MgO. Furthermore, good thermodynamic descriptions provide phase diagrams and thermodynamic data that can also effectively provide a theoretical basis for material design [2,3,4,5,6].

Xie et al. [7] utilized the modified quasi-chemical model to describe the liquid phase and firstly optimized the thermodynamic parameters of the P_2_O_5_-Na_2_O system based on the reliable experimental phase diagram and thermodynamic properties, and their calculations were in good agreement with the experimental data, while the description of the enthalpy of formation of Na_5_P_3_O_10_ was inaccurate. In 2015, Ding et al. [8] evaluated a P_2_O_5_-MgO system by using the modified quasi-chemical model to describe the liquid phase, and PO_4_^3−^ was considered the basic unit of P_2_O_5_ in the liquid phase, but the calculations showed significant discrepancies with the experimental data. Furthermore, a set of thermodynamic data describing the liquid phase with the modified quasi-chemical model does not simultaneously describe both the oxide and metal liquid phases, which limits the study of the phosphorus distribution ratio between liquid iron and oxide slags. Therefore, it is meaningful to construct a set of multicomponent thermodynamic databases that can describe both oxide and metal liquids using appropriate thermodynamic models to guide the addition of oxide fluxes in the steelmaking dephosphorization process. The ionic two-sublattice model allowing one set of the thermodynamic parameters to simultaneously describe both the oxide and metallic liquid [9] was used to describe the liquid phase for the first time in the current work. Additionally, the ionic two-sublattice model can not only rationally describe the phosphate melt structure but also adequately reproduce the thermodynamic properties of complex liquids such as slag [10,11]. This is highly beneficial to the construction of a slag system multivariate database to guide steelmaking dephosphorization.

This work aimed to conduct a phase diagram thermodynamic optimization of the P_2_O_5_-Na_2_O and P_2_O_5_-MgO systems using the CALPHAD (CAlculation of PHAse Diagram) approach through establishing suitable thermodynamic models. The crystal structure, limited measured phase diagram and thermodynamic properties were optimized to construct a Gibbs energy expression for each phase in the systems to obtain a set of thermodynamic parameters reasonably describing the phase diagrams, covering the whole composition range using Thermo-Calc software.

## 2. Review of Literature Data

The experimental phase diagram information and thermodynamic property data of the P_2_O_5_-Na_2_O and P_2_O_5_-MgO systems are systematically evaluated. The crystal structures of the solid phases in the systems are listed in Table 1.

### 2.1. P_2_O_5_-Na_2_O System

A phase diagram of the P_2_O_5_-Na_2_O system has been reported by several researchers [34,35,36,37,38,39]. Partridge et al. [34], using thermal, microscopic and X-ray diffraction (XRD) analysis, determined the liquidus of the NaPO_3_-Na_4_P_2_O_7_ system and confirmed the presence of the Na_5_P_3_O_10_ compound. Two invariant reactions of L = β − NaPO_3_ + α − Na_5_P_3_O_10_ and L + α − Na_4_P_2_O_7_ = α − Na_5_P_3_O_10_ were reported to occur at 824 K and 893 K, respectively. And the melting points of NaPO_3_, Na_5_P_3_O_10_ and Na_4_P_2_O_7_ were 898 K, 788 K and 1258 K, respectively. In their work [34], the Na_4_P_2_O_7_ and NaPO_3_ phases exhibited a lot of phase transitions from room temperature to melting point. The transition temperatures of Na_4_P_2_O_7_ were found to be 673 K, 783 K, 793 K and 818 K by differential thermal analysis (DTA). Two phase transitions of NaPO_3_ at 677 K and 783 K were detected. Subsequently, Morey and Ingerson [35] also studied the phase equilibria of the NaPO_3_-Na_4_P_2_O_7_ system in good agreement with the work of Partridge et al. [34]. Two invariant reactions L = β − NaPO_3_ + α − Na_5_P_3_O_10_ and L + α − Na_4_P_2_O_7_ = α − Na_5_P_3_O_10_ were measured to have reaction temperatures of 825 K and 895 K, and the melting points of NaPO_3_ and Na_4_P_2_O_7_ were observed to be 901 K and 1262 K, respectively, but the third structure of NaPO_3_ was not found. Turkdogan et al. [36], using the thermal, microscopic, and DTA methods, determined the phase diagram of the NaPO_3_-Na_3_PO_4_ system and did not report the presence of Na_5_P_3_O_10_. Three invariant reaction temperatures of L = β − NaPO_3_ + α − Na_5_P_3_O_10_, L + α − Na_4_P_2_O_7_ = α − Na_5_P_3_O_10_ and L = α − Na_4_P_2_O_7_ + β − Na_3_PO_4_ in the NaPO_3_-Na_3_PO_4_ system were suggested to be 763 K, 893 K and 1218 K by Markina et al. [37], respectively. In 1970, Osterheld et al. [38] determined the phase transition temperature of the Na_4_P_2_O_7_-Na_3_PO_4_ system below 1573 K by thermal analysis and high-temperature microscopy. They reported that the eutectic reaction L = α − Na_4_P_2_O_7_ + β − Na_3_PO_4_ occurred at 1225 K, and two compounds (Na_4_P_2_O_7_ and Na_3_PO_4_) melted congruently at 1271 K and 1785 K, respectively. In 1972, Berak et al. [39] observed three invariant reactions in the liquidus study of the Na_2_O-P_2_O_5_ system. The liquidus data obtained from these works for the P_2_O_5_-Na_2_O system were in reasonable agreement and were used in the optimization process of the current work. The four compounds NaPO_3_, Na_5_P_3_O_10_, Na_4_P_2_O_7_ and Na_3_PO_4_ have polymorphic phase transitions, and the thermodynamic description of the phase transition of the compounds in the P_2_O_5_-Na_2_O system by Xie et al. [7] based on the reliable literature is more complete, which was considered in the thermodynamic assessment of the present work with refinement and improvement. It is worth noting that there is less information about the experimental phase relation of the P_2_O_5_-rich and Na_2_O-rich regions in the P_2_O_5_-Na_2_O system, which still needs to be further determined experimentally.

In 1909, Mixter [40] determined the enthalpy of formation of NaPO_3_ from its elements using solution calorimetry (SCA). In 1967, Irving et al. [41] also utilized SCA to measure the enthalpy of formation of Na_3_PO_4_ from its elements at 298 K. Subsequently, in 1968, Irving et al., [42,43] Krivtsov et al., [44] and Zhuang et al. [45] determined the enthalpies of formation of Na_4_P_2_O_7_, Na_5_P_3_O_10_ and NaPO_3_ from their elements using the SCA method. In 2011, Khaled et al. [46] measured the standard enthalpy of formation of Na_4_P_2_O_7_ from its elements using the SCA method. These experimental results were incorporated into the present study with consideration for possible error margins. Andon et al. [47] determined the heat capacities of NaPO_3_, Na_5_P_3_O_10_, Na_4_P_2_O_7_ and Na_3_PO_4_ using adiabatic calorimetry within the temperature range of 10 to 320 K. Ashcroft et al. [48] measured the heat capacities of Na_4_P_2_O_7_ and NaPO_3_ from 298 to 620 K and determined the low-temperature transition enthalpy of Na_4_P_2_O_7_. Lazarev et al. [49] used DSC to measure the heat capacity of Na_4_P_2_O_7_ in the temperature range from 300 to 1000 K and measured the low-temperature enthalpy of transition of the Na_4_P_2_O_7_. Grantscharova et al. [50] used DSC to determine the heat capacity of NaPO_3_ between 468 and 675 K, but their measurements were much higher than those reported by Ashcroft et al. [48]. Considering the above-reported heat capacity data, the data reported by Andon et al. [47], Ashcroft et al. [48] and Lazarev et al. [49] were considered in the present work to optimize the heat capacities of Na_4_P_2_O_7_ and NaPO_3_.

### 2.2. P_2_O_5_-MgO System

The phase diagram of the P_2_O_5_-MgO system in the composition ranges from 0 to 50 mol.% P_2_O_5_ was investigated by Berak [51] using thermal, microscopy and XRD analyses. In this concentration range, three intermediate compounds were observed: Mg_3_P_2_O_8_, Mg_2_P_2_O_7_ and MgP_2_O_6_ with melting points at 1630 K, 1655 K and 1438 K, respectively. These phases were considered as line compounds. The temperature of three eutectic reactions L = MgO + Mg_3_P_2_O_8_, L = Mg_3_P_2_O_8_ + α − Mg_2_P_2_O_7_ and L = α − Mg_2_P_2_O_7_ + MgP_2_O_6_ were found to be 1598 K, 1555 K and 1423 K, respectively. Additionally, Mg_3_P_2_O_8_ with two polymorphic forms was confirmed, and its transition temperature was 1328 K. Subsequently, Bobrownicki and Slawski [52] also measured the melting temperature of Mg_3_P_2_O_8_ to be 1628 K and the structural transition temperature to be 1323 K. However, these two studies did not give data such as the lattice parameter and the structural transition of Mg_3_P_2_O_8_, which have not been reported in subsequent studies [53,54]. Therefore, the optimization process of the present work did not consider the phase transformation of Mg_3_P_2_O_8_. Bookey [55], using thermal analysis, investigated the eutectic reaction L = MgO + Mg_3_P_2_O_8_ by means of cooling curves, which yielded a reaction temperature of 1603 K. The results were consistent with the data reported by Berak [51]. The melting points of Mg_3_P_2_O_8_ and Mg_2_P_2_O_7_ were investigated, and the presence of the phase transition in the Mg_2_P_2_O_7_ was determined by Czupinska et al. [53] and Oetting et al. [54] using thermal analysis. Combined with the data obtained by Roy et al. [56] and Calvo et al. [57], only the structural transformation of the Mg_2_P_2_O_7_ in the low-temperature region was considered in the present work. MgP_4_O_11_ was reported to melt congruently at 1183 K by Meyer et al. [32] using DTA. Rakotomahanina-Rolaisoa et al. [58] investigated the melting point of MgP_2_O_6_ by DTA.

In 1897, Berthelot [59] determined the enthalpy of formation of Mg_3_P_2_O_8_ from elements using SCA. In 1952, Bookey et al. [55] investigated the enthalpy of formation of Mg_3_P_2_O_8_. In 1954, the enthalpy of formation of Mg_3_P_2_O_8_ from elements was measured by Stevens and Turkdogan [60] using SCA. In 1986, Lopatin et al. [61] studied the standard enthalpies of formation of Mg_2_P_2_O_7_ and MgP_2_O_6_ from elements using the Knudsen cell mass spectrometry (KCMS) approach. In 1989, Lopatin et al. [62] used the KCMS method to determine the enthalpy of formation of Mg_3_P_2_O_8_ from elements. In 1999, Abdelkader et al. [63] measured the standard enthalpy of formation of Mg_3_P_2_O_8_ from elements using the SCA approach. These experimental data on the enthalpies of formation of the compounds in the P_2_O_5_-MgO system described above were accepted for the present work. Oetting and Mcdonald [54] measured the heat capacities of Mg_3_P_2_O_8_ and Mg_2_P_2_O_7_ using an adiabatic calorimeter and determined the heat contents of Mg_3_P_2_O_8_ and Mg_2_P_2_O_7_ in the temperature range from 0 to 1700 K. Furthermore, the energy change in the low-temperature phase transition of Mg_2_P_2_O_7_ was determined. Iwase et al. [64] investigated the activity of P_2_O_5_ in liquid P_2_O_5_-MgO mixtures using solid oxide galvanic cell techniques at 1673 K. Given that the reported data were obtained from indirect calculations, the data on the activity were not used in the current work.

## 3. Thermodynamic Modeling

The CALPHAD method is used to formulate a comprehensive thermodynamic model to describe each phase in a system, drawing upon experimental data encompassing phase diagrams, thermodynamic properties and crystal structures. This method rationally selects undetermined parameters to represent each phase of the system as a Gibbs free energy function of variables such as temperature, pressure and composition. Ultimately, the phase diagrams and thermodynamic properties are derived through the utilization of a thermodynamic database containing these Gibbs free energy expressions. In the present study, the thermodynamic assessment of the P_2_O_5_-Na_2_O and P_2_O_5_-MgO systems will be conducted using Thermo-Calc software. Employing the least-squares method, Thermo-Calc software endeavors to align the calculated values with the observed data, seeking optimized variable values that minimize the sum of squared differences between calculated and experimental data. Hence, the formulation of an appropriate thermodynamic model lays the groundwork for an excellent thermodynamic database.

The following thermodynamic models were used to model the P_2_O_5_-Na_2_O and P_2_O_5_-MgO systems in the present work. The constructed thermodynamic models used for two binary systems are listed in Table 2 and will be described below in more detail.

### 3.1. Pure Unary Component

The Gibbs energy *G_i_*(*T*) of pure unary component *i* can be expressed as follows:

(1) $$G_{i}\left(T\right)-H_{i}^{SER}=a+bT+cTlnT+dT^{2}+eT^{-2}+fT^{3}+gT^{7}+hT^{-9}$$

where *H_i_^SER^* is the standard molar enthalpy of pure unary component *i* at 298.15 K and 101,325 Pa, J·mol^−1^; *a*~*h* are the parameters to be optimized; *T* is the thermodynamic temperature, K.

### 3.2. Liquid Phase

In the current assessment, the ionic two-sublattice model is used to describe the liquid phase of the P_2_O_5_-Na_2_O and P_2_O_5_-MgO systems. The ionic two-sublattice model assumes that cations only mix with each other, and anions only mix with each other. This model comprises two sublattices: one for cations and the other for anions, neutrals, and vacancies.

In the liquid phase of the P_2_O_5_-Na_2_O and P_2_O_5_-MgO systems, the content of anions such as PO_3_^−1^, P_2_O_7_^−4^, PO_4_^−3^ varies with the composition of the system oxides [8]. To simplify the thermodynamic model by reducing the thermodynamic parameters, only the two anions (PO_3_^−1^ and PO_4_^−3^) are considered in the optimized modeling process. Therefore, the thermodynamic models of the liquid phase of the P_2_O_5_-Na_2_O and P_2_O_5_-MgO systems are (Na^+1^)_P_(O^−2^, PO_3_^−1^, PO_4_^−3^, PO_5/2_)_Q_ and (Mg^+2^)_P_(O^−2^, PO_3_^−1^, PO_4_^−3^, PO_5/2_)_Q_, where P and Q denote the total valence of the anion sublattice and the total valence of the cation sublattice, respectively. To maintain the electroneutrality of the liquid phase of the systems, the stoichiometric factors P and Q are allowed to change with the composition of the system oxides. Taking the P_2_O_5_-Na_2_O system as an example, the Gibbs energy of the liquid phase is expressed as follows:

(2) GmLiquid−HiSER=yNa+1yO−2GNa+1:O−2Liquid+yNa+1yPO3−1GNa+1:PO3−1Liquid+yNa+1yPO4−3GNa+1:PO4−3Liquid+Q(yPO5/2ln⁡yPO5/2)+PRTyNa+1ln⁡yNa+1+QRTyO−2ln⁡yO−2+yPO3−1ln⁡yPO3−1+yPO4−3ln⁡yPO4−3+yPO5/2ln⁡yPO5/2+GmLiquid E

where *H_i_^SER^* is the molar enthalpy of the pure unary component in the reference state of the standard element at 298.15 K and 101,325 Pa, J·mol^−1^; *y* is the site fraction of each species in the liquid phase in their respective sublattices; *G* is the Gibbs energy for the formation of the end-member, J·mol^−1^; *R* is the gas constant (R = 8.314 J·(mol·K)^−1^); GmLiquid E is the excess Gibbs energy, J·mol^−1^, which is denoted as follows:

(3) GmLiquid E=yNa+1yO−2yPO3−1LNa+1:O−2,PO3−1Liquid 0+yNa+1yO−2yPO4−3(LNa+1:O−2,PO4−3Liquid 0+LNa+1:O−2,PO4−3Liquid 1(yO−2−yPO4−3)           +LNa+1:O−2,PO4−3Liquid 2yO−2−yPO4−32)+yNa+1yO−2yPO5/2LNa+1:O−2,PO5/2Liquid 0           +yNa+1yPO3−1yPO4−3(LNa+1:PO3−1 ,PO4−3Liquid 0+LNa+1:PO3−1 ,PO4−3Liquid 1(yPO3−1−yPO4−3)           +LNa+1:PO3−1 ,PO4−3Liquid 2yPO3−1−yPO4−32+LNa+1:PO3−1 ,PO4−3Liquid 3yPO3−1−yPO4−33)           +yNa+1yPO3−1yPO5/2(LNa+1:PO3−1,PO5/2Liquid 0+LNa+1:PO3−1 ,PO5/2Liquid 1(yPO3−1−yPO5/2)           +LNa+1:PO3−1 ,PO5/2Liquid 2yPO3−1−yPO5/22)+yNa+1yPO4−3yPO5/2LNa+1:PO4−3 ,PO5/2Liquid 0

where *^i^L^Liquid^*(*i* = 0, 1, 2, 3) represents the interaction of the species in each sublattice and is the interaction parameter to be optimized.

### 3.3. Intermediate Compounds

In this work, all solid phases Na_3_PO_4_, Na_4_P_2_O_7_, Mg_3_P_2_O_8,_ Mg_2_P_2_O_7_, etc., are described as stoichiometric compounds. For the solid phase with heat capacity data, the Gibbs free energy *G_m_* is expressed as follows:

(4) $$G_{m}-H^{SER}=a+bT+cTlnT+dT^{2}+eT^{-1}$$

where *H^SER^* is the molar enthalpy of the pure elements (Na, Mg, P and O) in the reference state of the standard element at 298.15 K and 101,325 Pa, J·mol^−1^; *a*~*e* are the parameters which will be optimized; *T* is the thermodynamic temperature, K.

For the solid phase lacking heat capacity data, taking the P_2_O_5_-Na_2_O system as an example, the Gibbs energy ^0^*G_m_* of the solid is expressed as follows:

(5) Gm 0−HSER=xGNa2ONa2O_γ 0+yGP2O5P2O5_H 0+A+BT

where *x* and *y* are the ratios of Na_2_O and P_2_O_5_ in the solid phase; GNa2ONa2O_γ 0 and GP2O5P2O5_H 0 are the Gibbs energy of the solid phase of Na_2_O and P_2_O_5_, respectively, J·mol^−1^; *A* and *B* are the parameters which will be evaluated.

## 4. Results and Discussion

The thermodynamic optimization of the P_2_O_5_-Na_2_O and P_2_O_5_-MgO binary systems was carried out based on the critical evaluation of phase equilibrium and thermodynamic property data using Thermo-Calc software in the current study. During the optimization process, certain emphasis was given to each dataset of phase equilibrium and thermodynamic property data, taking into account their reliability. By adjusting the thermodynamic parameters for each phase within the systems, the calculated results could reasonably describe the experimental data within the acceptable error range.

Initially, the solid-phase parameters were optimized using experimental data, encompassing the heat capacity and formation enthalpy of the intermediate phases. Subsequently, liquid-phase parameters were incorporated to replicate the liquidus and invariant reactions of the systems. Finally, all parameters were simultaneously optimized by considering all reliable experimental data to obtain a set of thermodynamic parameters capable of effectively describing the P_2_O_5_-Na_2_O and P_2_O_5_-MgO binary systems, as presented in Table 2.

### 4.1. P_2_O_5_-Na_2_O System

The Gibbs energy functions for the components P_2_O_5_ and Na_2_O were sourced from the works of Jung et al. [65] and Wu et al. [66], respectively. Initially, the heat capacities of polymorphic forms of Na_4_P_2_O_7_ and NaPO_3_ were determined by fitting experimental data from Andon et al. [47], Ashcroft et al. [48] and Lazarev et al. [49], which were treated as identical within this study. Subsequently, the formation enthalpies of the four intermediate phases were optimized using experimental data on formation enthalpies from elements [40,41,42,43,44,45,46]. Then, the liquid parameters such as LNa+1:PO3−1,PO4−3Liquid 0, LNa+1:PO3−1,PO4−3Liquid 1, etc., were adjusted to replicate the liquidus and invariant reactions of the P_2_O_5_-Na_2_O binary system. Finally, all parameters were optimized simultaneously by considering all available experimental data.

**Figure 1:**
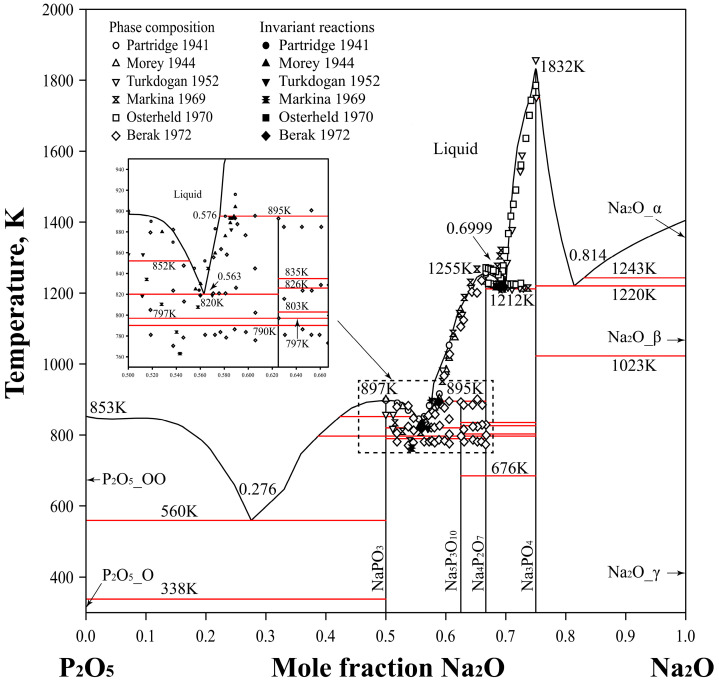
Calculated phase diagram of the P_2_O_5_-Na_2_O binary system compared with the experimental data [34,35,36,37,38,39].

Figure 1 presents the optimized phase diagram of the P_2_O_5_-Na_2_O binary system in comparison with experimental data [34,35,36,37,38,39]. The eutectic reactions L = β − NaPO_3_ + α − Na_5_P_3_O_10_ and L = α − Na_4_P_2_O_7_ + β − Na_3_PO_4_ are calculated to occur at temperatures of 820 K and 1212 K, respectively, while the peritectic reaction L + α − Na_4_P_2_O_7_ = α − Na_5_P_3_O_10_ takes place at 895 K. The difference in the calculated X(Na_2_O) from the experimentally determined values reported in the literature is less than 0.9 mol.%, marking a substantial improvement over the calculation of Xie et al. [7] and aligning more closely with the experimental data. Due to the limited availability of liquidus data for the P_2_O_5_-Na_2_O system, the predicted temperatures for the L = γ − NaPO_3_ + O’ − P_2_O_5_ and L = β − Na_3_PO_4_ + β − Na_2_O reactions calculated in this study are 560 K and 1220 K, respectively, which warrant validation through further experiments. The calculated liquidus points generally correspond well with the experimental data in the literature. Table 3 provides a comparison of the calculated invariant reactions involving the liquid phase in the P_2_O_5_-Na_2_O binary system with experimental data. It is evident that the calculated results of this study can effectively describe most of the available experimental information.

**Figure 2:**
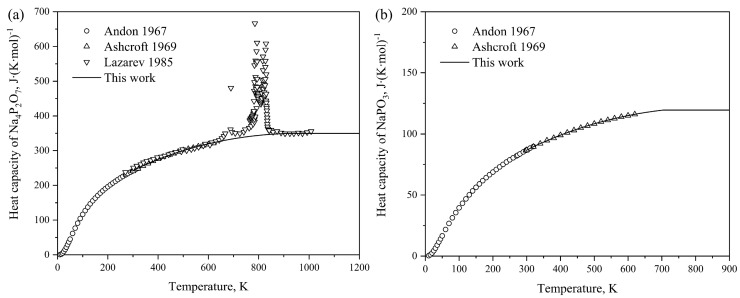
Calculated heat capacities of Na_4_P_2_O_7_ (**a**) and NaPO_3_ (**b**) compared with the experimental data [47,48,49].

**Figure 3:**
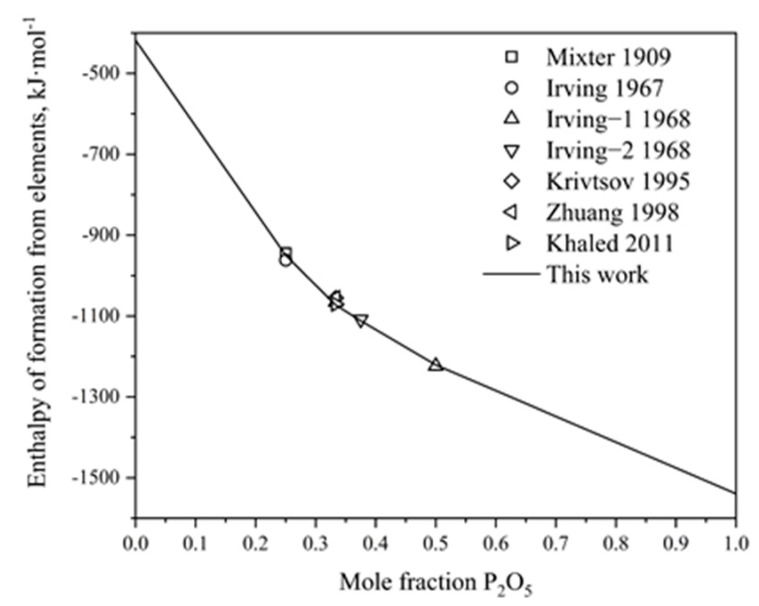
Calculated enthalpies of formation for the intermediate compounds of the P_2_O_5_-Na_2_O binary system at 298.15 K from elements compared with the experimental data [40,41,42,43,44,45,46].

Utilizing the optimized thermodynamic parameters, the thermodynamic properties of the P_2_O_5_-Na_2_O system are computed. Figure 2a and b show the calculated heat capacities of Na_4_P_2_O_7_ and NaPO_3_, respectively, obtained in this study, juxtaposed with experimental data measured by Andon et al. [47], Ashcroft et al. [48] and Lazarev et al. [49]. The calculated results exhibit satisfactory agreement with the measured values. For Na_5_P_3_O_10_ and Na_3_PO_4_, the Neumann–Kopp equation was employed to describe their heat capacities due to the limited experimental data available. The standard enthalpies of formation of the intermediate compounds from elements (BCC_A2 for sodium and white phosphorus) at 298 K are also calculated in this work, as depicted in Figure 3. The graph illustrates that our calculated results are generally consistent with the experimental values from Refs. [40,41,42,43,44,45,46]. Considering experimental uncertainties, the calculations are deemed acceptable.

### 4.2. P_2_O_5_-MgO System

**Figure 4:**
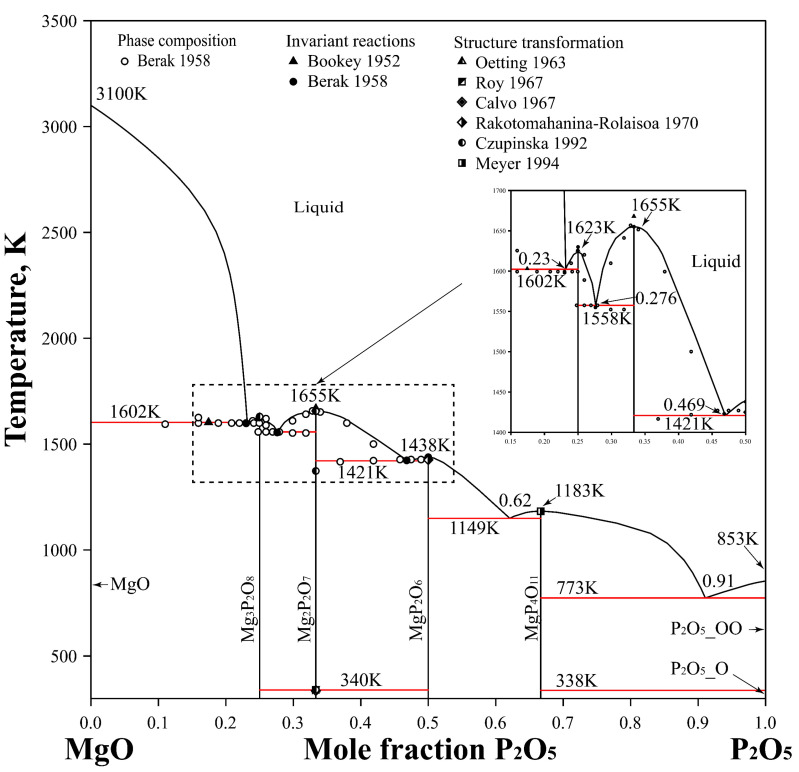
Calculated phase diagram of the P_2_O_5_-MgO binary system compared with the experimental data [32,51,53,54,55,56,57,58].

The Gibbs energy functions for the components P_2_O_5_ and MgO utilized in this study were sourced from Jung et al. [65] and Mao et al. [67], respectively. The heat capacities of Na_4_P_2_O_7_ and NaPO_3_ were modeled using experimental data from Oetting et al. [54]. In the present research, it is assumed that the heat capacities of both allotropic forms of Mg_2_P_2_O_7_ were equal. The optimized phase diagram of the P_2_O_5_-MgO system, presented in Figure 4, is compared with experimental data [32,51,53,54,55,56,57,58]. Additionally, the temperature and phase composition details of invariant reactions are juxtaposed with the experimental data reported in the literature, as shown in Table 4. It is evident that the calculated phase boundaries align well with the experimental information found in the literature. The present study provides a better and more reasonable description of the experimental data for the P_2_O_5_-MgO system compared to the results of Ding et al. [8].

The phase relationship in the composition range above 50 mol.% P_2_O_5_ remains to be definitively determined experimentally, owing to the limited available experimental data. In the optimization process, two eutectic reactions were predicted in this portion of the phase diagram. The calculated reaction temperatures are 1149 K for the reaction L = MgP_2_O_6_ + MgP_4_O_11_ and 773 K for the reaction L = MgP_4_O_11_ + O’ − P_2_O_5_. Correspondingly, the calculated X(P_2_O_5_) values are 62 mol.% and 91 mol.%, respectively.

**Figure 5:**
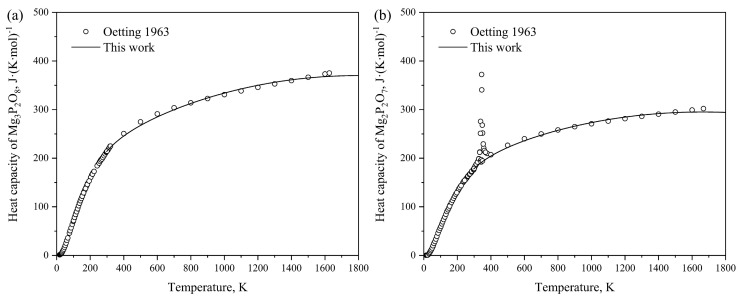
Calculated heat capacities of Mg_3_P_2_O_8_ (**a**) and Mg_2_P_2_O_7_ (**b**) compared with the experimental data [54].

**Figure 6:**
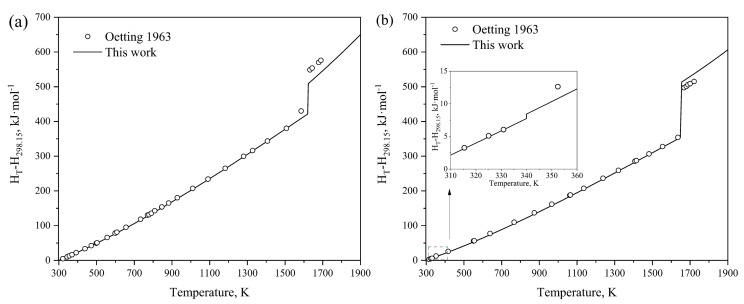
Calculated heat contents of Mg_3_P_2_O_8_ (**a**) and Mg_2_P_2_O_7_(**b**) compared with the experimental data [54].

**Figure 7:**
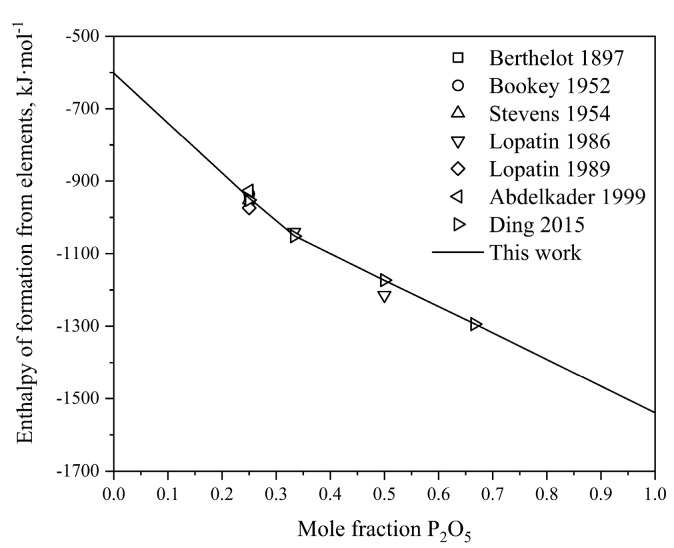
Calculated enthalpies of formation for the intermediate compounds of the P_2_O_5_-MgO binary system at 298.15 K from elements compared with the experimental data [55,59,60,61,62,63] and calculated results from the literature [8].

The heat capacities of Mg_3_P_2_O_8_ and Mg_2_P_2_O_7_ obtained by optimization in this work are illustrated in Figure 5a,b. Reasonable agreement is obtained between our calculated results and the heat capacities of Mg_3_P_2_O_8_ and Mg_2_P_2_O_7_ in the temperature range from 298.15 K to 1800 K determined by Oetting et al. [54]. To describe the heat capacities of MgP_2_O_6_ and MgP_4_O_11_, the Neumann–Kopp equation was employed during the optimization process to sum the heat capacities of Na2O_γ and P2O5_H. Figure 6a,b present the calculated heat contents of Mg_3_P_2_O_8_ and Mg_2_P_2_O_7_ based on the obtained thermodynamic parameters, compared with the experimental data [54]. The results indicate a close alignment with the experimental values, with acceptable deviations considering experimental errors. The calculated melting enthalpy of Mg_3_P_2_O_8_($∆H_{melt}$ = 97.449 kJ·mol^−1^) is slightly lower than the experimental value reported by Oetting et al. [54], while the calculated melting enthalpy of Mg_2_P_2_O_7_($∆H_{melt}$ = 160.03 kJ·mol^−1^) is slightly higher than the experimental value. This brings the calculated values much closer to the experimental results compared to the study by Ding et al. [8]. Additionally, the calculated enthalpy of transition from Mg_2_P_2_O_7__β to Mg_2_P_2_O_7__α at 340 K is determined to be 0.68 kJ·mol^−1^. Figure 7 shows the calculated standard enthalpies of formation for the intermediate compounds from elements at 298 K compared with the experimental data [55,59,60,61,62,63] and calculated results from the literature [8]; the reference states are the Mg of HCP_A3 and white phosphorus, which reproduce the standard enthalpy of formation for the compounds from elements very well. As can be seen, a precise description of the experimental thermodynamic properties of the system can be provided by utilizing the calculated thermodynamic parameters within the acceptable margin of error.

## 5. Conclusions

The CALPHAD method was utilized to critically evaluate and assess the P_2_O_5_-Na_2_O and P_2_O_5_-MgO binary systems. The main conclusions are summarized below:

1. A set of self-consistent thermodynamic parameters is derived for the P_2_O_5_-Na_2_O and P_2_O_5_-MgO binary systems based on a critical evaluation of the available phase diagram and thermodynamic property data. The calculated phase diagrams and thermodynamic properties employing the obtained thermodynamic parameters well reproduce the data reported in the literature.
2. In comparison with the previous assessments using the modified quasi-chemical model for the liquid phase, the present study using the ionic two-sublattice model to express the liquid phase for the first time can describe the experimental data of the P_2_O_5_-Na_2_O and P_2_O_5_-MgO binary systems in a better and more reasonable way, particularly the invariant reactions involving the liquid phase. The difference in the phase composition and temperature of invariant reactions from the experimentally determined values reported in the literature is less than 0.9 mol.% and 5K, respectively.
3. Four eutectic reactions (L = γ − NaPO_3_ + O’-P_2_O_5_, L = β − Na_3_PO_4_ + β − Na_2_O, L = MgP_2_O_6_+ MgP_4_O_11_ and L = MgP_4_O_11_ + O’ − P_2_O_5_) are predicted in the P_2_O_5_-Na_2_O and P_2_O_5_-MgO binary systems. The predicted temperatures of these eutectic reactions are 560 K, 1220 K, 1149 K and 773 K, with the corresponding phase compositions X(P_2_O_5_) being 82.4 mol%, 18.6 mol%, 62 mol% and 91 mol%, respectively. These predictions await further experimental validation.

## Figures and Tables

**Table 1:** Crystal structures of all solid phases in the P_2_O_5_-Na_2_O and P_2_O_5_-MgO systems.

System	Compound	Crystal System	Space Group	Reference
P_2_O_5_-Na_2_O	γ-NaPO_3_	Orthorhombic	*P*2_1_*P*2_1_*P*2*_1_*	[12]
		Orthorhombic	*Pnma*	[13]
	β-NaPO_3_	Triclinic	*P*2_1_/*n*	[14]
	α-NaPO_3_	Monoclinic	*P*2_1_/*c*	[15]
	β-Na_5_P_3_O_10_	Monoclinic	*C*2/*c*	[16]
	α-Na_5_P_3_O_10_	Monoclinic	*C*2/*c*	[17]
	α-Na_4_P_2_O_7_	Orthorhombic	*P*2_1_*P*2_1_*P*2_1_	[18]
	β-Na_3_PO_4_	Tetragonal	*P*$\overset{-}{4}$2_1_*c*	[19]
	α-Na_3_PO_4_	Cubic	*Fm* $\overset{-}{3}$ *m*	[20]
		Orthorhombic	*Pnma*	[21]
P_2_O_5_-MgO	Mg_3_P_2_O_8_	Monoclinic	*P*2_1_/*b*	[22]
		Monoclinic	*P*2_1_/*n*	[23]
		Monoclinic	*P*2_1_/*n*	[24]
		Monoclinic	*P*2_1_/*n*	[25]
		Triclinic	*P* $\overset{-}{1}$	[26]
	β-Mg_2_P_2_O_7_	Monoclinic	*P*2_1_/*c*	[27]
	α-Mg_2_P_2_O_7_	Monoclinic	*C*2/*m*	[28]
	MgP_2_O_6_	Monoclinic	*C*2/*c*	[29]
		Monoclinic	*C*2/*c*	[30]
	MgP_4_O_11_	Monoclinic	*P*2_1_/*c*	[31]
		Monoclinic	*P*2_1_/*c*	[32]
		Orthorhombic	*Pmc*2_1_	[33]

α/β/γ: the polymorph from high temperature to low temperature.

**Table 2:** The obtained thermodynamic parameters of the P_2_O_5_-Na_2_O and P_2_O_5_-MgO systems in the present work.

System	Phase	Formula	Thermodynamic Parameter/J·mol^−1^
P_2_O_5_-Na_2_O	Liquid	(Na^+1^)_p_(O^−2^, PO_3_^−1^, PO_4_^−3^, PO_5/2_)_q_	GNa+1:O−2Liquid 0=+GNa2OLiquid 0
			GPO5/2Liquid 0=+GP2O5Liquid 0
			GNa+1:PO3−1Liquid 0=+0.5GNa2OLiquid 0+0.5GP2O5Liquid 0−223581.5−46.8T
			GNa+1:PO4−3Liquid 0=+1.5GNa2OLiquid 0+0.5GP2O5Liquid 0−597241+62T
			LNa+1:PO3−1,PO4−3Liquid 0=−127756+18T
			LNa+1:PO3−1,PO4−3Liquid 1=−63351
			LNa+1:O−2,PO4−3Liquid 0=+7424
			LNa+1:PO3−1,PO5/2Liquid 0=−48065
			LNa+1:PO3−1,PO5/2Liquid1=−37884
	Na_3_PO_4__β	(Na^+1^)_3_(P^+5^)_1_(O^−2^)_4_	GNa+1:P+5:O−2Na3PO4_α 0=+GNa3PO4Solid 0
	Na_3_PO_4__α	(Na^+1^)_3_(P^+5^)_1_(O^−2^)_4_	GNa+1:P+5:O−2Na3PO4_β 0=+GNa3PO4Solid 0+472−0.27T
	Na_4_P_2_O_7__ζ	(Na^+1^)_4_(P^+5^)_2_(O^−2^)_7_	GNa+1:P+5:O−2Na4P2O7_α 0=+GNa4P2O7Solid 0
	Na_4_P_2_O_7__ε	(Na^+1^)_4_(P^+5^)_2_(O^−2^)_7_	GNa+1:P+5:O−2Na4P2O7_β 0=+GNa4P2O7Solid 0+10040−14.65693431T
	Na_4_P_2_O_7__δ	(Na^+1^)_4_(P^+5^)_2_(O^−2^)_7_	GNa+1:P+5:O−2Na4P2O7_γ 0=+GNa4P2O7Solid 0+13806−19.38215388T
	Na_4_P_2_O_7__γ	(Na^+1^)_4_(P^+5^)_2_(O^−2^)_7_	GNa+1:P+5:O−2Na4P2O7_δ 0=+GNa4P2O7Solid 0+15061−20.94504305T
	Na_4_P_2_O_7__β	(Na^+1^)_4_(P^+5^)_2_(O^−2^)_7_	GNa+1:P+5:O−2Na4P2O7_ε 0=+GNa4P2O7Solid 0+17153−23.47773070T
	Na_4_P_2_O_7__α	(Na^+1^)_4_(P^+5^)_2_(O^−2^)_7_	GNa+1:P+5:O−2Na4P2O7_ζ 0=+GNa4P2O7Solid 0+20082−26.98551513T
	Na_5_P_3_O_10__β	(Na^+1^)_5_(P^+5^)_3_(O^−2^)_10_	GNa+1:P+5:O−2Na5P3O10_α 0=+GNa5P3O10Solid 0
	Na_5_P_3_O_10__α	(Na^+1^)_5_(P^+5^)_3_(O^−2^)_10_	GNa+1:P+5:O−2Na5P3O10_β 0=+GNa5P3O10Solid 0+10878−13.769620T
	NaPO_3__γ	(Na^+1^)_1_(P^+5^)_1_(O^−2^)_3_	GNa+1:P+5:O−2NaPO4_α 0=+GNaPO3Solid 0
	NaPO_3__β	(Na^+1^)_1_(P^+5^)_1_(O^−2^)_3_	GNa+1:P+5:O−2NaPO4_β 0=+GNaPO3Solid 0+628−0.78795483T
	NaPO_3__α	(Na^+1^)_1_(P^+5^)_1_(O^−2^)_3_	GNa+1:P+5:O−2NaPO4_γ 0=+GNaPO3Solid 0+4226−5.010959525T
P_2_O_5_-MgO	Liquid	(Mg^+2^)_p_(O^−2^, PO_3_^−1^, PO_4_^−3^, PO_5/2_)_q_	GMg+2:O−2Liquid 0=+2GMgOLiquid 0
			GPO5/2Liquid 0=+GP2O5Liquid 0
			GMg+2:PO3−1Liquid 0=+GMgOLiquid 0+GP2O5Liquid 0−238484−10T
			GMg+2:PO4−3Liquid 0=+3GMgOLiquid 0+GP2O5Liquid 0−709347+185T
			LMg+2:PO3−1,PO4−3Liquid 0=−128900+45T
			LMg+2:PO3−1,PO4−3Liquid 1=+13365
			LMg+2:PO3−1,PO4−3Liquid 2=−16547
			LMg+2:PO3−1,PO4−3Liquid 3=+40441
			LMg+2:O−2,PO4−3Liquid 0=−57701
			LMg+2:O−2,PO4−3Liquid 1=+6541
			LMg+2:O−2,PO4−3Liquid 2=−8954
			LMg+2:PO3−1,PO5/2Liquid 0=−92054+60T
			LMg+2:PO3−1,PO5/2Liquid 1=−25546
			LMg+2:PO3−1,PO5/2Liquid 2=−35451
			LMg+2:O−2,PO3−1Liquid 0=−66748+51T
	Mg_3_P_2_O_8_	(Mg^+2^)_3_(P^+5^)_2_(O^−2^)_8_	GMg+2:P+5:O−2Mg3P2O8 0=+GMg3P2O8Solid 0
	Mg_2_P_2_O_7__β	(Mg^+2^)_2_(P^+5^)_2_(O^−2^)_7_	GMg+2:P+5:O−2Mg2P2O7_α 0=+GMg2P2O7Solid 0
	Mg_2_P_2_O_7__α	(Mg^+2^)_2_(P^+5^)_2_(O^−2^)_7_	GMg+2:P+5:O−2Mg2P2O7_β 0=+GMg2P2O7Solid 0+680−2.0T
	MgP_2_O_6_	(Mg^+2^)_1_(P^+5^)_2_(O^−2^)_6_	GMg+2:P+5:O−2MgP2O6 0=+GMgP2O6Solid 0
	MgP_4_O_11_	(Mg^+2^)_1_(P^+5^)_4_(O^−2^)_11_	GMg+2:P+5:O−2MgP4O11 0=+GMgP4O11Solid 0
Function	Temperature range/K		
GP2O5Liquid 0	(298.15–1000)		−1639225.067 − 230.7480381*T* + 21.643407*T*ln*T* − 0.1681142*T*^2^ + 1.87715×10^−5^*T*^3^ + 1758186.5*T*^−1^ + 22900.402ln*T*
	(1000–6000)		−1579441.75 + 1382.959261*T* − 225*T*ln*T*
GP2O5P2O5_OO 0	(298.15–1000)		−1665880.067 − 199.4980381*T* + 21.643407*T*ln*T* − 0.1681142*T*^2^ + 1.87715×10^−5^*T*^3^ + 1758186.5*T*^−1^ + 22900.402ln*T*
	(1000–6000)		−1606096.75 + 1414.209261*T* − 225*T*ln*T*
GP2O5P2O5_O 0	(298.15–1000)		−1666269.067 − 198.3480381*T* + 21.643407*T*ln*T* − 0.1681142*T*^2^ + 1.87715×10^−5^*T*^3^ + 1758186.5*T*^−1^ + 22900.402ln*T*
	(1000–6000)		−1606485.75 + 1415.359261*T* − 225*T*ln*T*
GP2O5P2O5_H 0	(298.15–1000)		−1631835.067 − 221.1390381*T* + 21.643407*T*ln*T* − 0.1681142*T*^2^ + 1.87715×10^−5^*T*^3^ + 1758186.5*T*^−1^ + 22900.402ln*T*
	(1000–6000)		−1572051.75 + 1392.568261*T* − 225*T*ln*T*
GNa2OLiquid 0	(298.15–1405)		−380898.2803 + 340.194781*T* − 66.216001*T*ln*T* − 0.021932551*T*^2^ + 2.34792×10^−6^*T*^3^ + 406685.01*T*^−1^
	(1405–1500)		−387789.21 + 580.2481164*T* − 104.6*T*ln*T*
GNa2ONa2O_α 0	(298.15–1405)		−428595.8803 + 374.143281*T* − 66.216001*T*ln*T* − 0.021932551*T*^2^ + 2.34792×10^−6^*T*^3^ + 406685.01*T*^−1^
	(1405–1500)		−435486.81 + 614.1966164*T* − 104.6*T*ln*T*
GNa2ONa2O_β 0	(298.15–1405)		−440520.2803 + 383.736481*T* − 66.216001*T*ln*T* − 0.021932551*T*^2^ + 2.34792×10^−6^*T*^3^ + 406685.01*T*^−1^
	(1405–1500)		−447411.21 + 623.7898164*T* − 104.6*T*ln*T*
GNa2ONa2O_γ 0	(298.15–1405)		−442277.5603 + 385.454281*T* − 66.216001*T*ln*T* − 0.021932551*T*^2^ + 2.34792×10^−6^*T*^3^ + 406685.01*T*^−1^
	(1405–1500)		−449168.49 + 625.5076164*T* − 104.6*T*ln*T*
GNa3PO4Solid 0	(298.15–6000)		+GNa2ONa2O_γ 0 + 0.5GP2O5P2O5_H 0 − 520688.946 − 4.353*T*
GNa4P2O7Solid 0	(298.15–967)		−3282062.70195 + 815.816308*T* − 145.08494*T*ln*T* − 0.215875*T*^2^ + 3.77148×10^−5^*T*^3^ + 542554.7741*T*^−1^
	(967–1273)		−3322276.2378 + 2062.32142*T* − 349.82659*T*ln*T*
GNa5P3O10Solid 0	(298.15–6000)		+2.5GNa2ONa2O_γ 0 + 1.5GP2O5P2O5_H 0 − 1144087.404 − 20.487*T*
GNaPO3Solid 0	(298.15–703)		−1243133.8886 + 264.3713457*T* − 46.08288*T*ln*T* − 0.09082*T*^2^ + 1.80447×10^−5^*T*^3^ + 188542.1015*T*^−1^
	(703–973)		−1256643.34549 + 704.6628499*T* − 119.50568*T*ln*T*
GMgOLiquid 0	(298.15–1700)		−549098.33 + 275.724634*T* − 47.4817*T*ln*T* − 0.00232681*T*^2^ +4.5043×10^−8^*T*^3^ + 516900*T*^−1^
	(1700–2450)		−585159.646 + 506.06825*T* − 78.3772*T*ln*T* + 0.0097344*T*^2^ −8.60338×10^−7^*T*^3^ + 8591550*T*^−1^
	(2450–3100)		+9110429.75−42013.7634*T* + 5298.548*T*ln*T* − 1.30122485*T*^2^ + 5.8262601×10^−5^*T*^3^ − 3.24037416×10^9^*T*^−1^
	(3100–5100)		−632664.468 + 589.239555*T* − 84*T*ln*T*
GMgOSolid 0	(298.15–1700)		−619428.502 + 298.253571*T* − 47.4817*T*ln*T* − 0.00232681*T*^2^ +4.5043×10^−8^*T*^3^ + 516900*T*^−1^
	(1700–3100)		−655489.818 + 528.597187*T* − 78.3772*T*ln*T* + 0.0097344*T*^2^ −8.60338×10^−7^*T*^3^ + 8591550*T*^−1^
	(3100–5000)		−171490.159–1409.43369*T* + 163.674142*T*ln*T* − 0.044009535*T*^2^ + 1.374896×10^−6^*T*^3^−1.72665403×10^8^*T*^−1^
	(5000–5100)		−722412.718 + 617.657452*T*−84*T*ln*T*
GMg3P2O8Solid 0	(298.15–1800)		−3863914.664 + 1191.277265*T* − 195.04422*T*ln*T*−0.098665*T*^2^ + 9.22527×10^−6^*T*^3^ + 1562390.321*T*^−1^
GMg2P2O7Solid 0	(298.15–1800)		−3217696.802 + 1003.594497*T* − 165.99611*T*ln*T* − 0.07885*T*^2^ + 7.96388×10^−6^*T*^3^ + 1371185.587*T*^−1^
GMgP2O6Solid 0	(298.15–6000)		+GMgOSolid 0 + GP2O5P2O5_H 0 − 240840 + 2.95*T*
GMgP4O11Solid 0	(298.15–6000)		+GMgOSolid 0 + GP2O5P2O5_H 0 – 269030 − 4.29*T*

**Table 3:** Calculated invariant reactions involving the liquid phase in the P_2_O_5_-Na_2_O binary system.

Reaction	Type	Liquid Composition/Mole Fraction Na_2_O	Temperature/K	Reference
L = γ − NaPO_3_ + O’ − P_2_O_5_	Eutectic	0.276	560	This work
L = β − NaPO_3_ + α − Na_5_P_3_O_10_	Eutectic	0.559	824	[34]
		0.556	825	[35]
		0.57	819	[36]
		0.543	763	[37]
		0.56	819	[39]
		0.56	833	[7]
		0.563	820	This work
L + α − Na_4_P_2_O_7_ = α − Na_5_P_3_O_10_	Peritectic	0.587	893	[34]
		0.588	895	[35]
		0.585	893	[37]
		0.589	893	[39]
		0.575	898	[7]
		0.576	895	This work
L = α − Na_4_P_2_O_7_ + β − Na_3_PO_4_	Eutectic	0.684	1218	[37]
		0.694	1225	[38]
		0.6975	1217	[39]
		0.691	1209	[7]
		0.6999	1212	This work
L = β − Na_3_PO_4_ + β − Na_2_O	Eutectic	0.814	1220	This work

**Table 4:** Calculated invariant reactions involving the liquid phase in the P_2_O_5_-MgO binary system.

Reaction	Type	Liquid Composition/Mole Fraction P_2_O_5_	Temperature/K	Reference
L = MgO + Mg_3_P_2_O_8_	Eutectic	0.23	1598	[51]
		-	1603	[55]
		0.23	1596	[8]
		0.23	1602	This work
L = Mg_3_P_2_O_8_ + α − Mg_2_P_2_O_7_	Eutectic	0.276	1555	[51]
		0.277	1563	[8]
		0.276	1558	This work
L = α − Mg_2_P_2_O_7_ + MgP_2_O_6_	Eutectic	0.468	1423	[51]
		0.469	1410	[8]
		0.469	1421	This work
L = MgP_2_O_6_ + MgP_4_O_11_	Eutectic	0.62	1149	This work
L = MgP_4_O_11_ + O’ − P_2_O_5_	Eutectic	0.91	773	This work

## Data Availability

The original contributions presented in the study are included in the article, further inquiries can be directed to the corresponding authors.

## References

[1] Suito H., Inoue R. (1984). Effects of Na_2_O and BaO additions on phosphorus distribution between CaO-MgO-FetO-SiO_2_-slags and liquid iron. Trans. Iron Steel Inst. Jpn..

[2] Chen T., Yuan Y., Wang J., Wu J., Wang B., Chen X., Moelans N., Wang J., Pan F. (2024). Features and classification of solid solution behavior of ternary Mg alloys. J. Magnes. Alloys.

[3] Chen T., Gao Q., Yuan Y., Li T., Xi Q., Liu T., Tang A., Watson A., Pan F. (2021). Coupling physics in machine learning to investigate the solution behavior of binary Mg alloys. J. Magnes. Alloys.

[4] Yi W., Liu G., Gao J., Zhang L. (2021). Boosting for concept design of casting aluminum alloys driven by combining computational thermodynamics and machine learning techniques. J. Mater. Inf..

[5] Zhang S., Yi W., Zhong J., Gao J., Lu Z., Zhang L. (2022). Computer alloy design of Ti modified Al-Si-Mg-Sr casting alloys for achieving simultaneous enhancement in strength and ductility. Materials.

[6] Tian Y., Jiang K., Deng Z., Wang K., Zhang H., Liu L., Zhang L. (2023). Integration of CALPHAD calculations and nanoindentation test for the design of low-modulus near-β titanium. J. Cent. South Univ..

[7] Xie W., Wei S.H., Hudon P., Jung I., Qiao Z., Cao Z. (2020). Critical evaluation and thermodynamic assessment of the R_2_O-P_2_O_5_ (R = Li, Na and K) systems. Calphad.

[8] Ding G., Xie W., Jung I., Qiao Z., Du G., Cao Z. (2015). Thermodynamic assessment of the MgO-P_2_O_5_ and CaO-P_2_O_5_ Systems. Acta Phys.-Chim. Sin..

[9] Zhang L., Gao F., Deng T., Liu Y., Yang L., Guo C., Tan J., Ma T., Chen W., Du Y. (2022). Phase equilibria in the FeO-Fe_2_O_3_-SiO_2_ system: Experimental measurement and thermodynamic modeling. Calphad.

[10] Yang L., Zeng Y., Guo C., Liu Y., Li B., Deng T., Chen W., Du Y. (2024). Experimental investigation and thermodynamic assessment of the Na_2_O-Al_2_O_3_-CaO system. Ceram. Int..

[11] Zhang L., Liu Y., Gao F., Tan J., Yang L., Deng T., Chen W., Ouyang Y., Du Y. (2024). Thermodynamic description of the FeO-Fe_2_O_3_-MgO system and its extrapolation to the X-MgO-FeO-Fe_2_O_3_ (X= CaO and SiO_2_) systems. J. Am. Ceram. Soc..

[12] Wiench D.M., Jansen M. (1983). Untersuchungen an Tetranatrium-cyclo-tetraphosphat(V) und seinen Hydraten. Monatsh. Chem..

[13] Ondik H.M. (1965). The structure of anhydrous sodium trimetaphosphate Na_3_P_3_O_9_, and the monohydrate, Na_3_P_3_O_9_. H_2_O. Acta Crystallogr..

[14] Jost K.H. (1963). Die Struktur des Kurrol’schen Na-Salzes (NaPO_3_)_x_, Typ B. Acta Crystallogr..

[15] Corbridge D.E.C. (1955). Crystallographic data on some Kurrol salts. Acta Crystallogr..

[16] Dymon J.J., King A.J. (1951). Structure studies of the two forms of sodium tripolyphosphate. Acta Crystallogr..

[17] Corbridge D.E.C. (1960). The crystal structure of sodium triphosphate, Na_5_P_3_O_10_, phase I. Acta Crystallogr..

[18] Leung K.Y., Calvo C. (1972). The Structure of Na_4_P_2_O_7_ at 22 °C. Can. J. Chem..

[19] Lissel E., Jansen M., Jansen E., Will G. (1990). Bestimmung der Kristallstruktur von T-Na_3_PO_4_ mit Rontgen- und Neutronenpulvertechniken. Z. Für Krist..

[20] Newsan J.M., Cheetham A.K., Tofield B.C. (1980). Structural studies of the high-temperature modifications of sodium and silver orthophosphates, II-Na_3_PO_4_ and II-Ag_3_PO_4_, and of the low-temperature form I-Ag_3_PO_4_. Solid State Ion..

[21] Kizilyalli M., Welch A.J.E. (1976). Preparation and X-ray powder diffraction data for anhydrous sodium orthophosphates. J. Inorg. Nucl. Chem..

[22] Berthet G., Joubert J.C., Bertaut E.F. (1972). Vacancies ordering in new metastable orthophosphates [Co_3_□]P_2_O_8_ and [Mg_3_□]P_2_O_8_ with olivin-related structure. Z. Für Krist..

[23] Nord A.G., Kierkegaard P. (1968). The crystal structure of Mg_3_(PO_4_)_2_. Acta Chem. Scand..

[24] Baykal A., Kizilyalli M., Kniep R. (1997). Synthesis and Characterisation of Anhydrous Magnesium Phosphate Mg_3_(PO_4_)_2_. Turk. J. Chem..

[25] Nord A.G., Stefanidis T. (1980). The cation distribution between five-and six-coordinated sites in some (Mg, Me)_3_(PO_4_)_2_ solid solutions. Mater. Res. Bull..

[26] Jaulmes S., Elfakir A., Quarton M., Brunet F., Chopin C. (1997). Structure cristalline de la phase haute température et haute pression de Mg_3_(PO_4_)_2_. J. Solid State Chem..

[27] Lukaszewicz K. (1967). Crystal structure of alpha-Mg_2_P_2_O_7_ and the mechanism of phase transition beta->alpha-Mg_2_P_2_O_7_. Bull. Acad. Pol. Sci., Ser. Sci. Chim..

[28] Datars W.R. (1967). ESR Study of Mn^2+^ in α- and β-Mg_2_P_2_O_7_. J. Chem. Phys..

[29] Beucher M., Grenier J.C. (1968). Donnees cristallographiques sur les tetrametaphosphates du type MII_2_P_4_O_12_ (MII=Ni, Mg, Zn, Cu, Co, Mn). Mater. Res. Bull..

[30] Nord A.G., Lindberg K.B. (1975). The Crystal Structure of Magnesium Tetrametaphosphate, Mg_2_P_4_O_12_. Acta Chem. Scand..

[31] Stachel D., Paulus H., Guenter C., Fuess H. (1992). Crystal structure of magnesium ultraphosphate, MgP_4_O_11_. Z. Für Krist..

[32] Meyer K., Hobert H., Barz A., Stachel D. (1994). Infrared spectra and structure of various crystalline ultraphosphates and their glasses. Vib. Spectrosc..

[33] Yakubovich O.V., Dimitrova O.V., Vidrevich A.I. (1993). Magnesium ultraphosphate MgP_4_O_11_: Growth and crystal structure. Crystallogr. Rep..

[34] Partridge E.P., Hicks V., Smith G.W. (1941). A Thermal, Microscopic and X-Ray Study of the System NaPO_3_-Na_4_P_2_O_7_^1^. J. Am. Chem. Soc..

[35] Morey G.W., Ingerson E. (1944). The binary system NaPO_3_-Na_4_P_2_O_7_. Am. J. Sci..

[36] Turkdogan E.T. (1952). Phase Equilibrium Investigation of the Na_2_O-P_2_O_5_-SiO_2_ Ternary System. J. Iron Steel Inst. Lond..

[37] Markina I.B., Voskresenskaya N.K. (1969). Fusibility of a mutual system of sodium and potassium meta- and orthophosphates. Russ. J. Inorg. Chem..

[38] Osterheld R.K., Bahr E.W. (1970). Liquidus diagram for the sodium orthophosphate-sodium pyrophosphate system. J. Inorg. Nucl. Chem..

[39] Berak J., Znamierowska T. (1972). Phase equilibria in the system CaO-Na_2_O-P_2_O_5_. Part II. The partial system Ca(PO_3_)_2_-Na_2_O-P_2_O_5_. Rocz. Chem..

[40] Mixter W.G. (1909). ART. XII-The Heat of Formation of Trisodium Ortho phosphate, Trisodium Orthoarsenate, the Oxides of Antimony, Bismuth Trioxide; and fourth paper on the Heat of Combination of Acidic Oxides with Sodium Oxide. Am. J. Sci..

[41] Irving R.J., McKerrell H. (1967). Standard heats of formation of NaH_2_PO_4_, Na_2_HPO_4_ and Na_3_PO_4_. Trans. Faraday Soc..

[42] Irving R.J., McKerrell H. (1968). Standard heats of formation of two sodium pyrophosphates, sodium trimetaphosphate, and sodium tetrametaphosphate. Trans. Faraday Soc..

[43] Irving R.J., McKerrell H. (1968). Standard heats of formation of the sodium triphosphates Na_5_P_3_O_10_(cI), Na_5_P_3_O_10_(cII) and Na_5_P_3_O_10_.6H_2_O(c). Trans. Faraday Soc..

[44] Krivtsov N.V., Titova K.V., Rosolovskii V.Y. (1995). The Enthalpy of Dissolution and Standard Enthalpy of Formation of Sodium Pyrophosphate Peroxosolvate Na_4_P_2_O_7_∙3H_2_O_2_. Russ. J. Inorg. Chem..

[45] Zhuang W., Liang J., Qiao Z., Shen J., Shi Y., Rao G. (1998). Estimation of the standard enthalpy of formation of double oxide. J. Alloys Compd..

[46] Khaled H.G.B., Khattech I., Jemal M. (2011). Standard enthalpy of formation of disodium hydrogen phosphate hexahydrate and sodium diphosphate. J. Chem. Thermodyn..

[47] Andon R.J.L., Counsell J.F., Martin J.F., Mash C.J. (1967). Thermodynamic properties of phosphorus compounds. II. Low-temperature heat capacity and entropy of sodium mono-, di-, and tri-phosphates. J. Appl. Chem..

[48] Ashcroft S.J., Keen E., Mortimer C.T. (1969). Thermochemistry of formation of sodium polyphosphates from sodium orthophosphates. Trans. Faraday Soc..

[49] Lazarev V.B., Sokolova I.D., Sharpataya G.A. (1985). DSC study op polymorphism of Na_4_P_2_O_7_. Thermochim. Acta.

[50] Grantscharova E., Avramov I., Gutzow I. (1986). Calorimetric study of vitreous and crystalline sodium metaphosphate NaPO_3_. Thermochim. Acta.

[51] Berak J. (1958). The system magnesium oxide-phosphorus pentoxide. Rocz. Chem..

[52] Bobrownicki W., Slawski K. (1959). Pseudobinary section Ca_3_(PO_4_)_2_-Mg_3_(PO_4_)_2_ in the ternary system CaO-MgO-P_2_O_5_. Rocz. Chem..

[53] Czupinska G. (1992). The system YPO4-Mg_3_(PO_4_)_2_-Mg_2_P_2_O_7_. J. Therm. Anal..

[54] Oetting F.L., McDonald R.A. (1963). The thermodynamic properties of magnesium orthophosphate and magnesium pyrophosphate. J. Phys. Chem..

[55] Bookey J.B. (1952). The free energy of formation of magnesium phosphate. J. Iron Steel Inst. Lond..

[56] Roy R., Middleswarth E.T., Hummel F.A. (1948). Mineralogy and thermal behavior of phosphates; I. Magnesium pyrophosphate. Am. Mineral..

[57] Calvo C. (1967). The crystal structure of α-Mg_2_P_2_O_7_. Acta Crystallogr..

[58] Rakotomahanina-Rolaisoa E., Henry Y., Durif A. (1970). Phase equilibrium diagram for TlPO_3_-Co(PO_3_)_2_, TlPO_3_-Mg(PO_3_)_2_, and TlPO_3_-Ca(PO_3_)_2_. Bull. Soc. Fr. Mineral. Cristallogr..

[59] Berthelot M. (1897). Thermochimie: Donnees et Lois Numériques.

[60] Stevens C.G., Turkdogan E.T. (1954). The heats of formation of trimanganous phosphate and trimagnesium phosphate. Trans. Faraday Soc..

[61] Lopatin S.I., Semenov G.A., Kutuzova Y.L.A. (1986). A mass-spectrometric investigation of the thermal dissociation of condensed magnesium phosphates. Inorg. Mater..

[62] Lopatin S.I., Semenov G.A. (1989). A Mass-spectrometric investigation of thermal dissociation of alkaline earth metal monophosphates. Neorg. Mater..

[63] Abdelkader S.B., Cherifa A.B., Khattech I., Jemal M. (1999). Synthèse, caractérisation et thermochimie du phosphate trimagnésien et du phosphate tricalcique. Thermochim. Acta.

[64] Iwase M., Akizuki H., Fujiwara H., Ichise E., Yamada N. (1987). A thermodynamic study of MgO-P_2_O_5_ slags by means of solid-oxide galvanic cell at 1673K. Steel Res..

[65] Jung I.H., Hudon P. (2012). Thermodynamic Assessment of P_2_O_5_. J. Am. Ceram. Soc..

[66] Wu P., Eriksson G., Pelton A.D. (1993). Optimization of the thermodynamic properties and phase diagrams of the Na_2_O-SiO_2_ and K_2_O-SiO_2_ systems. J. Am. Ceram. Soc..

[67] Mao H., Selleby M., Sundman B. (2004). A re-evaluation of the liquid phases in the CaO-Al_2_O_3_ and MgO-Al_2_O_3_ systems. Calphad.

